# The effect of a clinical decision support system on prompting an intervention for risky alcohol use in a primary care smoking cessation program: a cluster randomized trial

**DOI:** 10.1186/s13012-019-0935-x

**Published:** 2019-08-23

**Authors:** Nadia Minian, Dolly Baliunas, Aliya Noormohamed, Laurie Zawertailo, Norman Giesbrecht, Christian S. Hendershot, Bernard Le Foll, Jürgen Rehm, Andriy V. Samokhvalov, Peter L. Selby

**Affiliations:** 1 Nicotine Dependence Services, Centre for Addiction and Mental Health, 175 College St, Toronto, ON M5T1P7 Canada; 2 Department of Family and Community Medicine, University of Toronto, 500 University Ave, Toronto, ON M5G 1V7 Canada; 3 Dalla Lana School of Public Health, University of Toronto, 155 College, Toronto, ON M5T 3M7 Canada; 4 Department of Pharmacology and Toxicology, University of Toronto, 1 King’s College Cir, Toronto, ON M5S 1A8 Canada; 5 Institute for Mental Health Policy Research, Centre for Addiction and Mental Health, 33 Russell St, Toronto, ON M5S 2S1 Canada; 6 Campbell Family Mental Health Research Institute, Centre for Addiction and Mental Health, 60 White Squirrel Way, Toronto, ON M6J 1H4 Canada; 7 Department of Psychiatry, University of Toronto, 250 College Street, Toronto, ON M5T 1R8 Canada; 8 Institute of Medical Science, University of Toronto, 1 King’s College Cir, Toronto, ON M5S 3K1 Canada; 9 Institute for Clinical Psychology and Psychotherapy, TU Dresden, Chemnitzer Str. 46, 01187, Dresden, Germany

**Keywords:** Alcohol, Tobacco, Clinical decision support system, Cancer prevention, Interactive systems framework, Primary care

## Abstract

**Background:**

Clinical decision support systems (CDSSs) may promote practitioner adherence to evidence-based guidelines. This study examined if the addition of a CDSS influenced practitioner delivery of a brief intervention with treatment-seeking smokers who were drinking above recommended alcohol consumption guidelines, compared with practitioners who do not receive a CDSS prompt.

**Methods:**

This was a cluster randomized controlled trial conducted in primary health care clinics across Ontario, Canada, implementing the Smoking Treatment for Ontario Patients (STOP) smoking cessation program. Clinics randomized to the intervention group received a prompt when a patient reported consuming alcohol above the Canadian Cancer Society (CCS) guidelines; the control group did not receive computer alerts. The primary outcome was an offer of an appropriate educational alcohol resource, an alcohol reduction workbook for patients drinking above the CCS guidelines, and an abstinence workbook to patients scoring above 20 points in the AUDIT screening tool; the secondary outcome was patient acceptance of the resource. The tertiary outcome was patient abstinence from smoking, and alcohol consumption within CCS guidelines, at 6-month follow-up. Results were analyzed using a generalized estimation approach for fitting logistic regression using a population-averaged method.

**Results:**

Two hundred and twenty-one clinics across Ontario were randomized for this study; 110 to the intervention arm and 111 to the control arm. From the 15,222 patients that enrolled in the smoking cessation program, 15,150 (99.6% of patients) were screened for alcohol use and 5715 patients were identified as drinking above the CCS guidelines. No statistically significant difference between groups was seen in practitioner offer of an educational alcohol resource to appropriate patients (OR = 1.19, 95% CI 0.88–1.64, *p* = 0.261) or in patient abstinence from smoking and drinking within the CCS guidelines at 6-month follow-up (OR = 0.93, 95% CI 0.71–1.22, *p* = 0.594). However, a significantly greater proportion of patients in the intervention group accepted the alcohol resource offered to them by their practitioner (OR = 1.48, 95% CI 1.01–2.16, *p* = 0.045).

**Conclusion:**

A CDSS may not increase the likelihood of practitioners offering an educational alcohol resource, though it may have influenced patients’ acceptance of the resource.

**Trial registration:**

This trial is registered with ClinicalTrials.gov, number NCT03108144, registered on April 11, 2017, “retrospectively registered”.

**Electronic supplementary material:**

The online version of this article (10.1186/s13012-019-0935-x) contains supplementary material, which is available to authorized users.

Contributions to the literature

- This study used the Interactive Systems Framework to implement an electronic clinical decision support system (CDSS) to promote alcohol screening, brief intervention, and referral to treatment (SBIRT) in an Ontario-wide smoking cessation program.
- The intervention achieved large-scale implementation of SBIRT across 221 primary care sites.
- This large study augments an evolving literature and points to the complexities involved in the role of CDSS in clinical guideline implementation; the provision of a CDSS may not increase practitioner adherence to alcohol guidelines but may increase the likelihood that patients accept to receive an alcohol resource

Contributions to the literature

- This study used the Interactive Systems Framework to implement an electronic clinical decision support system (CDSS) to promote alcohol screening, brief intervention, and referral to treatment (SBIRT) in an Ontario-wide smoking cessation program.
- The intervention achieved large-scale implementation of SBIRT across 221 primary care sites.
- This large study augments an evolving literature and points to the complexities involved in the role of CDSS in clinical guideline implementation; the provision of a CDSS may not increase practitioner adherence to alcohol guidelines but may increase the likelihood that patients accept to receive an alcohol resource

## Background

Implementation science is focused on understanding and accelerating the integration of research findings and research-based innovation into everyday practice settings to improve health. This is critical given the substantial barriers between evidence-based knowledge and practice across all health care disciplines [1]. Health care practitioner decision-making is a highly complex and contextually dependent process. Non-adherence of practitioners to practice guidelines is common; there are intensive efforts by administrators and quality improvement specialists to promote evidence-based decision-making in a range of clinical settings [2]. Practitioner non-adherence has been identified as one of the most critical gaps impeding improvements in the health of people across the cancer control continuum [3]. For example, cancer risk due to dual tobacco and alcohol consumption can potentially be minimized by following recommended guidelines to provide screening, brief interventions, and referrals to treatment (SBIRT) to eligible patients in a primary care setting.

Although best-practice guidelines recommend treating tobacco and alcohol dependence concurrently to increase the chance of quit success for both substances [4, 5], treatment is often delivered separately [4–10], despite evidence of the multiplicative, positively associated risk for aero-digestive, and other related cancers resulting from the combined use of alcohol and tobacco [11–21].

Clinical decision support systems (CDSSs) have long been suggested as a way to promote practitioner adherence to evidence-based guidelines [22]. Researchers have shown the effectiveness of CDSS in improving prescribing practices [23–25], reducing medication errors [25, 26], improving preventive care (such as vaccination, smoking cessation, breast cancer screening) [27], and in ordering and recommending patients to clinical studies [25].

CDSS supports practitioners by synthesizing and integrating patient-specific information, performing complex evaluations, and presenting results in a timely fashion [28]. Given the increased use of electronic medical records in primary care offices, broader implementation of CDSS has become possible, offering an opportunity to improve evidence-based care.

While CDSS has been shown to be effective in improving health care processes across various clinical settings, it should be noted that over 30% of the studies assessed in a systematic review did not demonstrate improved clinical practice as a result of CDSS [25]. In addition, achieving large-scale adoption of CDSS remains a challenge [29].

This cluster randomized controlled trial aimed to assess whether the addition of a web-based CDSS—designed to prompt practitioners in real time to conduct SBIRT with patients who are drinking above recommended alcohol consumption guidelines—influences the probability of practitioners delivering a brief intervention to their eligible patients attempting to quit smoking compared to those practitioners who did not receive a prompt during the clinical encounter. A cluster randomized trial, with clinics as the units of randomization and patients as the unit of analysis, was chosen to prevent contamination that would result if practitioners working within a clinic were exposed to both arms of the trial. This trial—addressing *com*bined *a*lcohol and *t*obacco use—will be referred to as COMBAT throughout this paper.

## Methods

### Implementation framework

The Interactive Systems Framework (ISF) for Dissemination and Implementation [30] guided the implementation of this study. ISF includes three systems that need to interact with one another to implement scientific knowledge: the synthesis and translation system, the support system, and the delivery system. As applied to this study, the synthesis and translation system consisted of an infographic, newsletters, and a slide deck that translated the latest research findings on the risks of alcohol and tobacco use into friendly formats for practitioners. As part of the support system, two 60-min-long interactive web-based SBIRT trainings delivered by Dr. Selby, the Director of Medical Education and Clinician Scientist at CAMH, were offered before the launch of the COMBAT trial both aiming to build the capacity of practitioners to deliver an alcohol intervention to their smoking patients. The recordings of these webinars can be seen at: https://adobe.ly/2XfXzMs and https://adobe.ly/2SeaR8D. All practitioners were invited to participate in the live webinars, and recordings of the webinars were shared with all practitioners. Primary health care clinics in Ontario, Canada, implementing an existing smoking cessation treatment program, the Smoking Treatment for Ontario Patients (STOP) program, are the delivery system. The STOP program is an established smoking cessation program implemented in health care organizations across Ontario, Canada. The STOP program offers up to 26 weeks of smoking cessation treatment, consisting of nicotine replacement therapy and behavioral counseling, at no cost to the patient. It is a real-world, pragmatic, smoking cessation service in which treatment is individually tailored to the patient by their STOP practitioner who can be a nurse, doctor, or social worker. Within a 1-year program of care, there is no mandated number of clinical care visits between practitioners and participants.

For this study, we examined if a CDSS would facilitate practitioners to deliver a brief alcohol intervention and an appropriate alcohol resource, an alcohol reduction workbook to their STOP patients drinking above the Canadian Cancer Society (CCS) guidelines (defined below) but below the AUDIT cutoff of 20 points, and alcohol abstinence resource to patients scoring above the AUDIT cutoff of 20 points. All brief interventions had to be given face to face by their STOP practitioner at the time they enrolled in the STOP program.

This study is reported according to recommended standards (see Additional files 1 and 2).

### Study design and setting

We conducted a pragmatic cluster randomized controlled trial (COMBAT), with parallel intervention and control groups, in primary health care clinics (clusters) in Ontario implementing the STOP program, between April 2016 and September 2017. At the time the COMBAT trial was implemented, 150 out of 184 family health teams (FHTs), 59 out of 67 community health centers (CHCs), and 17 out of 23 nurse practitioner-led clinics (NPLCs) in Ontario were delivering the STOP program and over 67,000 patients had enrolled by FHTs, CHCs, or NPLCs.

Practitioners can enroll patients in the STOP program using paper-based forms or a web-based data capture tool—the STOP portal. The STOP portal is a centralized system for recording and monitoring patient information, managing nicotine replacement inventory, and receiving real-time progress reports for the practice.

To be included in the COMBAT study, clinics had to be operational in the STOP program as of March 14, 2016, and use the STOP portal. Patients provided consent to enroll in the STOP program. The detailed protocol for this study has been published [31]. The study was approved by the Research Ethics Board of the Centre for Addiction and Mental Health.

### Clinic randomization

A study investigator (DB) stratified allocation assignments by clinic type (FHTs, CHCs, NPLCs) and predicted size (small, large) in a 1:1 allocation ratio to balance clinic-level characteristics between study arms. Actual clinic size was not known a priori therefore the predicted clinic size was estimated based on prior STOP enrollment; for each clinic type, two levels of clinic size were set such that the expected total enrollment in the two levels would be balanced. The allocation schedule was computer generated using statistical computer software Stata v13 [32]. Two study staff were un-blinded to allocation results so as to facilitate implementation of the random allocation sequence within the CDSS. Clinics were randomized en masse and, though not blinded to randomization assignment, were not informed of their allocation until the trial began. Specifically, clinics were informed that alcohol use was being addressed in the STOP study, but were not informed that some clinics were receiving a CDSS (which alerted practitioners of patients drinking above the CCS guidelines and guided them to provide an appropriate alcohol intervention and offer an appropriate alcohol resource) while others were not receiving the CDSS. All patients meeting patient-level eligibility criteria (described below) at enrollment during the study period were included in the study; patients were unaware of their study participation. Except for DB, who conducted the analysis, all investigators remained blinded until the last follow-up survey was completed. DB was blinded until the time of the analysis.

### Patient eligibility

Eligible patients were cigarette smokers who sought help via their clinic to quit smoking, reduce smoking, or maintain an existing quit attempt; who were drinking above Canadian Cancer Society (CCS) guidelines (defined below); and who enrolled in English, with their practitioner completing online (i.e., using the online portal) the enrollment procedures with them present.

### Treatment arms

#### Intervention arm

The intervention consisted of an online portal-embedded CDSS that (1) identified patients drinking above the CCS guidelines by automatically scoring and interpreting the results of the two evidence-based screening tools, a 7-day timeline followback (TLFB) questionnaire [33] and the Alcohol Use Disorders Identification Test (AUDIT) [34]. The CCS guidelines [18] recommend women drink less than one alcoholic beverage and men less than two alcoholic beverages a day. In the absence of explicit guidance on whether these guidelines refer to average consumption over a period of days or consumption on any given day, we interpreted the recommendation as being consistent with average consumption. Thus, patients drinking above the CCS guidelines were operationally defined as women consuming seven or more and men consuming 14 or more alcoholic beverages (13.6 g per standard drink) in the past week and/or any patients consuming five or more alcoholic beverages on one occasion; (2) prompted practitioners to provide a brief alcohol reduction or abstinence intervention; (3) prompted practitioners to provide a resource to stop drinking (to patients scoring above the AUDIT cutoff of 20 points) or reduce drinking (to patients drinking above the CCS guidelines but below the AUDIT cutoff). The alcohol reduction workbook was co-created with STOP patients and based on existing evidence-based resources [35]. The abstinence resource had provincial resources for the patient to help support abstaining from alcohol.

#### Control arm

Control clinics continued to use the online portal for enrollment of treatment-seeking smokers. They had access to the same screening questions and resources available to those in the intervention arm but did not receive computer alerts identifying patients who drank above guidelines, nor any guidance on which intervention to provide.

### Outcomes

The primary outcome of the study was the offer of an appropriate educational alcohol resource, matched to the severity on the AUDIT score reported in the STOP baseline enrollment survey—an alcohol reduction workbook for patients drinking above the CCS guidelines and an abstinence resource to patients scoring above the AUDIT cutoff of 20 points. A practitioner’s offer of an educational resource was measured dichotomously—yes or no—for each eligible patient in both control and intervention groups.

Given that patients can refuse practitioners’ offer of resources, the secondary outcome of the study measured the acceptance of an educational alcohol resource by patients exceeding alcohol use guidelines. Practitioners recorded whether the patient declined the resource in the online portal. The secondary outcome was also measured dichotomously—yes or no—for each eligible patient in both study arms.

The tertiary outcome was abstinence from smoking, and alcohol consumption within the CCS guidelines, measured via a 6-month follow-up survey sent to all eligible patients. Smoking abstinence was defined by a negative response to the question “Have you had a cigarette, even a puff, in the last 7 days?” Alcohol consumption at follow-up was measured using the same survey items as those used in the baseline survey (described previously).

### Sample size determination

Based on measures of alcohol consumption [36, 37] used in the STOP program prior to the start of the COMBAT trial, and accounting for intra-cluster correlation within each clinic as well as unequal enrollment by clinic, an enrollment of 23 participants per clinic was assumed over the 12-month course of this trial, in 189 clinics, for a total of 4347 subjects exceeding the CCS guidelines, with enrollment size varying by a coefficient of 1.04. This analysis set alpha = 0.05, power = 80%, and estimated an intra-cluster correlation coefficient of 0.04. Since there were no available estimates of the trial’s primary outcome measure prior to the initiation of the trial, the minimum detectable effect size was estimated for a range of values of *P*_1_, the proportion of control group enrollments in which practitioners delivered the brief intervention. The estimated detectable effect size ranged from a relative risk of 1.45 (if *P*_1_ = 0·1) to 1.04 (if *P*_1_ = 0·9).

An un-blinded third-party consultant conducted an interim analysis in the form of a power calculation 8 months following the study launch, once estimates of *P*1 (defined above) were available. As described above, a method was used that accounts for intra-cluster correlation within each clinic as well as unequal enrollment by clinic [38, 39]. The observed average cluster size was 30 and cluster sizes varied by a coefficient of 1.12, resulting in a design effect of 6.13. Setting alpha = 0.05, the consultant, un-blinded to treatment allocation, obtained an estimate of *P*_1_ from the data collected to that date. To detect a relative risk of 1.20, the required sample size was estimated to be 5308. Therefore, the recruitment period was extended to 17 months, when that sample size was achieved.

### Statistical methods

Descriptive statistics compared cluster-level and patient-level baseline characteristics between the two treatment groups. Results were analyzed using a generalized estimation approach for fitting logistic regression using a population-averaged method. Stratification variables were included as covariates. To account for clustering, an exchangeable correlation matrix and robust standard errors were specified [40]. The following self-reported baseline variables were treated as potential confounders: sex; age; body mass index; identifying as First Nations, Inuit, or Métis; household income; employment status; educational attainment; depression; heaviness of smoking index; Audit C score, marijuana use; opioid use; treatment for mental health problem; drug treatment; and comorbid conditions. None of the listed potential confounders changed the estimated treatment effect by at least 10% and therefore were not included in the final models. As the primary and secondary outcomes were captured by the online portal, there were no missing outcome data. The tertiary outcome was missing for 53% of participants; therefore, imputation was not conducted and complete case analyses were conducted for the purposes of hypothesis generation. Because the intra-cluster correlation coefficient was estimated to be negative, logistic regression without adjustment for clustering was used [41]. All analyses followed an intention-to-treat approach in which clusters were analyzed in the intervention arm to which they were originally assigned and participants that in which they enrolled.

## Results

**Fig. 1:**
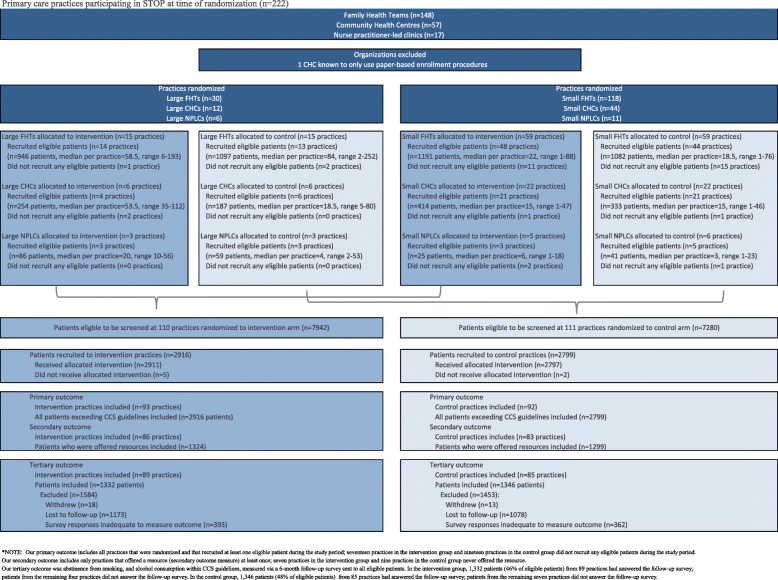
Number and types of practices who were enrolled, allocated, and analyzed in the COMBAT study. Our primary outcome includes all practices that were randomized and that recruited at least one eligible patient during the study period; seventeen practices in the intervention group and nineteen practices in the control group did not recruit any eligible patients during the study period. Our secondary outcome includes only practices that offered a resource (secondary outcome measure) at least once; seven practices in the intervention group and nine practices in the control group never offered the resource. Our tertiary outcome was abstinence from smoking, and alcohol consumption within the CCS guidelines, measured via a 6-month follow-up survey sent to all eligible patients. In the intervention group, 1332 patients (46% of eligible patients) from 89 practices had answered the follow-up survey; patients from the remaining four practices did not answer the follow-up survey. In the control group, 1346 patients (48% of eligible patients) from 85 practices had answered the follow-up survey; patients from the remaining seven practices did not answer the follow-up survey

Of the 222 practices (148 FHTs, 57 CHCs, and 17 NPLCs) that were assessed for eligibility, one practice was excluded because it was not using the STOP online portal. The remaining 221 practices were randomized: 110 practices (74 FHTs, 28 CHCs, and 8 NPLCs) were assigned to the intervention group, and 111 practices (74 FHTs, 28 CHCs, and 9 NPLCs) were assigned to the control group. Seventeen practices in the intervention group and 19 practices in the control group did not recruit any eligible patients during the study period (one site stopped recruiting STOP participants; 35 enrolled patients into STOP, but none were eligible for COMBAT); thus, data was only collected from 185 practices (93 intervention sites and 92 control sites). In total, 5715 patients drinking above the CCS guidelines were enrolled (Fig. 1). These patients comprise our main analytic sample. Figure 1 shows the number and types of practices who were enrolled, allocated, and analyzed in the COMBAT study.

Baseline characteristics for the individual and cluster level were similar in the intervention and the control groups (Table 1). Most patients had graduated high school, were daily smokers, and reported having an average of ten drinks containing alcohol in the week prior to their STOP enrollment. Approximately a third of patients had a household income below $40,000, which is below the low-income threshold for a family of Ontario’s average size of three [42]. Sites were located in all 14 local health integration networks (LHINs) across the province. Most sites had been implementing the smoking cessation program for at least 4 years prior to the launch of this study.

**Table 1:** Baseline patient and clinic characteristics for main analytic sample (*n* = 5715)

Variables	Intervention	Control
Individual level	(*n* = 2916)	(*n* = 2799)
Age in years (mean, SD)	47.8 (13.6)	48.1 (13.7)
Male	1600 (55)	1548 (55)
Graduated high school	2074 (71)	2014 (72)
Household income above 40 k	867 (30)	908 (32)
Currently employed	1523 (52)	1492 (53)
Daily smoking status	2751 (94)	2611 (93)
Heaviness of smoking index > 3	718 (26)	646 (25)
Number of alcohol drinks in past week (mean, SD)	10.6 (13.2)	10.5 (12.6)
Audit C score (med, IQR)	5 (4–7)	5 (4–7)
Audit 10 score > = 20	141 (5)	100 (4)
Past year attempts to quit smoking	1493 (51)	1432 (51)
Lifetime attempts to quit smoking > = 11	478 (16)	442 (16)
Marijuana use in past 30 days	968 (33)	852 (30)
Opioid use in past 30 days	435 (15)	419 (15)
Number of comorbid conditions endorsed (mean, SD)	2.5 (2.0)	2.3 (2.0)
Cluster level		
Participants per cluster (mean, SD)	31.4 (31.4)	30.4 (38.1)
Year clinic enrolled first patient in the STOP program		
2011	37 (40)	34 (37)
2012	32 (34)	26 (28)
2013	6 (6)	8 (9)
2014	9 (10)	13 (14)
2015	9 (10)	10 (11)
2016	0 (0)	1 (1)
Local Health Integration Networks* (health regions in Ontario)		
Central	5 (5)	4 (4)
Central East	7 (8)	9 (10)
Central West	3 (3)	2 (2)
Champlain	10 (11)	10 (11)
Erie-St. Clair	11 (12)	4 (4)
Hamilton Niagara Haldimand Brant	9 (10)	6 (7)
Mississauga Halton	1 (1)	2 (2)
North East	9 (10)	12 (13)
North Simcoe Muskoka	3 (3)	8 (9)
North West	5 (5)	5 (5)
South East	10 (11)	7 (8)
South West	8 (9)	10 (11)
Toronto Central	6 (6)	8 (9)
Waterloo Wellington	6 (6)	5 (5)

Note: Values are numbers (percentages) unless stated otherwise

*IQR* interquartile range, *SD* standard deviation

*Local Health Integration Networks (LHINs) are agencies established by the Government of Ontario to plan, coordinate, integrate, and fund health services at a local level. They represent health regions across the province. A total of 14 LHINs have been established across Ontario

Patient and clinic characteristics in each study arm are provided in Table 1.

As shown in Table 2, there was no significant difference between study arms in patients being offered an appropriate educational alcohol resource or in patients being abstinent from smoking and drinking within the CCS guidelines at the 6-month follow-up survey. However, a significantly greater proportion of patients in the intervention group accepted the educational alcohol resource offered by their practitioner than patients in the control group.

**Table 2:** Adjusted odds ratio and 95% confidence intervals for the primary, secondary, and tertiary outcomes

Outcomes	No. (%) in intervention group	No. (%) in control group	Intra-cluster correlation coefficient	Adjusted odds ratio (95% CI)	*p* value
Offer of appropriate resource	1324/2916 (45)	1254/2799 (45)	0.134	1.19 (0.88, 1.64)	0.261
Acceptance of offered resource*	280/1324 (21)	203/1299 (16)	0.074	1.48 (1.01, 2.16)	0.045
Abstinence from smoking and drinking within CCS guidelines	112/1332 (8)	121/1346 (9)	–	0.93 (0.71, 1.22)	0.594

Note: The first two outcomes, offer of appropriate resource and acceptance of offered resource, were derived from the electronic system and were measured at time of patient enrollment to the study while the third outcome, abstinence from smoking and drinking within the CCS guidelines, was derived from a patient questionnaire 6 months after enrollment

*Some practitioners offered an alcohol reduction resource to patients who should have received the abstinence resource and some practitioners offered an alcohol abstinence resource to patients who should have been offered an alcohol reduction resource. Since the secondary outcome was acceptance of the offered resource, whether appropriate or not appropriate, the numerator in the primary outcome and denominator in the secondary outcome do not match for the control group. In total, 45 participants in the control arm were offered an inappropriate resource

## Discussion

Providing primary care clinics offering a smoking cessation program with a CDSS did not result in an increase in eligible patients being offered an educational alcohol resource compared with usual care. However, providing clinics with a CDSS did result in an increase in the acceptance of an offered resource; patients were significantly more likely to accept the resource if their clinic had access to the CDSS. These results also provide compelling evidence that it is feasible (under the favorable conditions the STOP program provides) to achieve widespread adoption of a CDSS as well as to screen for alcohol use in primary care settings with smokers engaged in treatment for tobacco dependence; over 17 months, 99.6% of patients were screened for alcohol use and 45% of those who were drinking above the CCS guidelines were offered a brief intervention and an educational resource (i.e., referral to treatment). Compared to findings from a recently published study integrating SBIRT into primary care for alcohol abuse, among other substance use disorders and mental health conditions, alcohol screening in this trial was found to be high (56.8% vs 99.6%) [43]. Thus, the intervention was successful in integrating alcohol screening into primary care for alcohol use among smokers wanting to quit smoking.

Even though we developed the CDSS following the recommended features identified in a systematic review (the decision support was part of the routine workflow, a computer system was used to provide decision support, an explicit patient-specific recommendation was given, and the decision support was delivered at the time and location of decision-making) [44], our study did not find an effect of a CDSS on practitioners’ behavior (i.e., offering of the alcohol resource). This result is consistent with some previous studies; some systematic reviews of the effect of CDSS on practitioners’ behavior have found an effect [26, 28, 45–48] while others have not [49, 50]. The lack of effect in this study may reflect the health behavior that our CDSS targeted; several studies have shown that practitioners are not comfortable addressing alcohol with their patients [51, 52]. However, if practitioners decide to address alcohol, the CDSS might alter some contextual factor that impacted the likelihood of accepting the resource, helping explain the effect observed between CDSS and our secondary outcome. Unfortunately, the large proportion of non-response for the tertiary outcome measured on the patient follow-up survey limited our ability to answer the research question as intended but is presented nonetheless for the purpose of hypothesis generation. We did not find an association between the CDSS and patient smoking and drinking behavior at follow-up. This is consistent with prior studies [47], though these have been of small sample size and therefore were underpowered. Compared to findings from a recently published study, alcohol screening in this trial was found to be high (33% vs 99.6%) but providing a brief intervention was lower (54.8% vs 45%) [43].

Some limitations should be noted. The offer of an educational alcohol resource was only measured at a patient’s initial visit (which was for the purpose of enrolling in a smoking cessation program, not to receive help with the alcohol consumption); any offer that may have occurred during a future visit was not captured by the portal and reflect outcomes that this study is not able to address. In addition, our primary and secondary outcome relied on self-reports from practitioners, which might not be an accurate representation of actually proving a brief alcohol intervention and offering a resource. However, there is no reason to believe that this would be differential between study groups and therefore, if present, is unlikely to have biased the associations.

Although the primary outcome was recorded by the online portal, for seven patients, a malfunction occurred such that the practitioner completing the online enrollment procedures with the patient present was not presented with the option to provide take home alcohol reduction/abstinence resources to the patient, and these patients were treated as not having received an offer. However, this malfunction did not affect internal validity, as even if the malfunction was fully differential with respect to treatment arm, and the seven patients were either treated as having received an offer, or excluded entirely, the study results would not change substantively.

As noted in our protocol manuscript [31], the practitioner could potentially be employed at multiple clinics that may be assigned to different arms of this study (i.e., control and intervention). In this case, there is the possibility of contamination of knowledge; a practitioner might apply knowledge from the CDSS alerts in the intervention group to patients based at a control group clinic. This contamination could potentially compromise the effect of the trial, leading to a more conservative reporting estimate of the study’s overall effect.

Although all practitioners were offered online SBIRT training, we do not know which practitioners actually attended the training, and thus we cannot analyze the effect of the training on the offering of an educational alcohol resource. This analysis could have helped contextualize our results and offer further implementation recommendations.

Finally, our results may not be generalizable to other clinics seeking to implement similar CDSS since our study was pragmatic and occurred within the context of the STOP program. STOP program offers clinics with various resources (e.g., community of practice, ongoing operational support), which might impact the support system that the clinics received.

## Conclusions

This large study augments an evolving literature and shows that the provision of a CDSS may not increase practitioners’ offer of an educational alcohol resource and adherence to guidelines but may increase the likelihood of patients’ being receptive to accepting an alcohol resource. Identifying effective strategies that increase practitioners offering a brief intervention to smokers drinking above the CCS guidelines are still needed. Future studies could investigate strategies that increase practitioners’ adherence to SBIRT based on the CCS guidelines as well as identify contexts in which CDSS use is most effective. Based on the results of this study, future research could also examine if CDSS influence practitioner delivery of a brief intervention and offer of resources (e.g., persuasiveness of messaging) and if that consequently affects patient acceptance of an offer.

## Additional files

Additional file 1: CONSORT 2010 checklist of information to include when reporting a cluster randomised trial. (DOCX 36 kb)

Additional file 2: The TIDieR (Template for Intervention Description and Replication) Checklist. (DOCX 31 kb)

## Data Availability

The datasets generated and/or analyzed during the current study are not publicly available due to the fact that they contain personal health information, but are available from the corresponding author on reasonable request.

## Abbreviations

AUDIT: Alcohol Use Disorders Identification Test
CCS: Canadian Cancer Society
CDSS: Clinical decision support system
CHC: Community health centers
COMBAT: Personalized patient alerts and care pathways to prompt prevention interventions for combined alcohol and tobacco users in primary care
FHT: Family health team
ISF: Interactive Systems Framework
LHIN: Local health integration networks
NPLCs: Nurse practitioner-led clinics
SBIRT: Screening, brief intervention, and referral to treatment
STOP: Smoking Treatment for Ontario Patients
TLFB: Timeline followback
